# Sarcomere Formation Occurs by the Assembly of Multiple Latent Protein Complexes

**DOI:** 10.1371/journal.pgen.1001208

**Published:** 2010-11-18

**Authors:** Yanning Rui, Jianwu Bai, Norbert Perrimon

**Affiliations:** 1 Department of Genetics, Harvard Medical School, Boston, Massachusetts, United States of America; 2 Howard Hughes Medical Institute, Harvard Medical School, Boston, Massachusetts, United States of America; Stanford University, United States of America

## Abstract

The stereotyped striation of myofibrils is a conserved feature of muscle organization that is critical to its function. Although most components that constitute the basic myofibrils are well-characterized biochemically and are conserved across the animal kingdom, the mechanisms leading to the precise assembly of sarcomeres, the basic units of myofibrils, are poorly understood. To gain insights into this process, we investigated the functional relationships of sarcomeric protein complexes. Specifically, we systematically analyzed, using either RNAi in primary muscle cells or available genetic mutations, the organization of myofibrils in *Drosophila* muscles that lack one or more sarcomeric proteins. Our study reveals that the thin and thick filaments are mutually dependent on each other for striation. Further, the tension sensor complex comprised of zipper/Zasp/α-actinin is involved in stabilizing the sarcomere but not in its initial formation. Finally, integrins appear essential for the interdigitation of thin and thick filaments that occurs prior to striation. Thus, sarcomere formation occurs by the coordinated assembly of multiple latent protein complexes, as opposed to sequential assembly.

## Author Summary

Muscle functionality relies on the correct assembly of myofibrils, which are composed of tandem arrays of basic functional contractile units called the sarcomeres. Many mutations in genes encoding sarcomeric proteins cause muscle diseases such as congenital myopathy and dilated cardiac hypertrophy. Understanding the process of sarcomere assembly is not only relevant to the understanding of how protein complexes interact to form complex supra-molecular structures, but also of great significance to medicine for muscle diseases. Here, by taking advantage of our newly developed primary muscle cell culture method, we reevaluate sarcomere assembly by systematically analyzing the functional relationship of sarcomeric proteins using RNA interference or genetic ablation techniques. Our analysis leads us to propose a “two-state” model whereby sarcomeric proteins exist either in the “chaotic” state with independently assembled differential functional complexes or the “highly ordered suprastructure” state made from these complexes. Because we fail to detect any previously hypothesized sarcomere assembly intermediates in our system, our data support the model that sarcomere assembly is a highly coordinated process mediated by multiple latent protein complexes and does not occur in a step-wise fashion.

## Introduction

**Figure 1:**
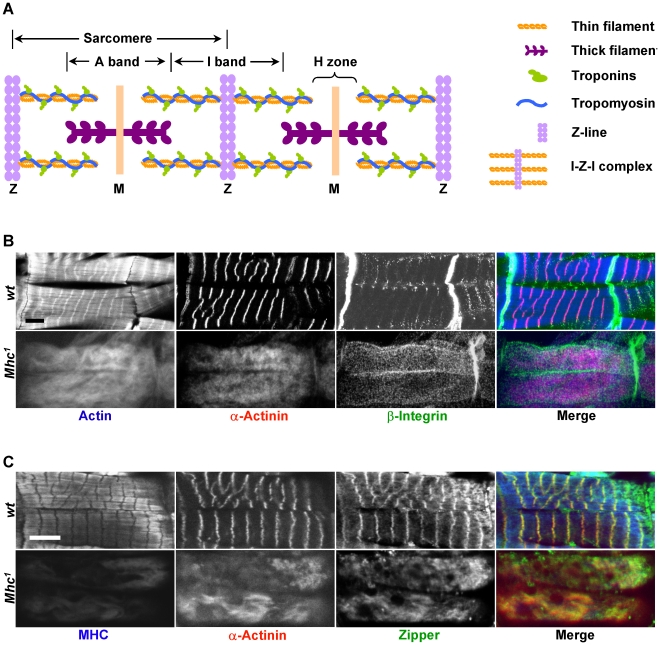
MHC is critical for muscle striation formation. (A) Schematic organization of a myofibril, represented here with two sarcomeres. Sarcomeres are defined as the segment between two neighboring Z-lines. Thin filaments include actin filaments and their associated proteins such as troponins (Tns) (TnC, TnI, and TnT) and tropomyosin (Tm). Actin filaments are the major components of I-bands, and are cross-linked to Z-lines via α-actinin. Thick filaments are composed of myosin and are connected from the M-line to the Z-line by titin. A number of proteins important for the stability of sarcomeres, such as zipper and Zasp, are found in the Z-line. (B) Confocal fluorescent micrographs of control muscles of a stage 17 wild-type embryo (top panels) and *myosin heavy chain (Mhc)* amorphic mutant muscles from *Mhc^1^* of same stage (bottom panels) stained by phalloidin (blue in merge), anti-α-actinin (red in merge) and anti-β-integrin (green in merge). Note that there is no obvious striation in *Mhc* null mutant muscles, and that β-integrin staining does not align with that of α-actinin. (C) Confocal images of control muscles of a stage 17 wild-type embryo (top panels) and *Mhc^1^* of same stage (bottom panels) stained by anti-muscle MHC (blue in merge), anti-α-actinin (red in merge) and anti-zipper (green in merge). Note that in wild-type muscles, zipper colocalizes with α-actinin as shown in yellow in the merged image, but not with MHC. In addition, rat-anti-MHC was able to detect truncated MHC fragments in *Mhc^1^* mutant muscles, and its staining overlaps with actin. This staining most likely reflects the ability of the Subfragment 1 region of MHC to bind to actin filaments. Scale bars: 10 µm.

Muscle functionality relies on the correct assembly of myofibrils, the cylindrical organelles attached to the cell surface membrane within muscle cells that run from one end of the cell to the other end. Myofibrils are composed of tandem arrays of basic functional contractile units called the sarcomeres. Sarcomeres are highly ordered, almost crystalline-like, structures composed of thin (actin) and thick (myosin) filaments and their associated proteins (Figure 1A). Although their components have been known for many years, how the various sarcomeric proteins assemble to form these highly ordered structures is poorly understood. Understanding the process of sarcomere assembly is not only relevant to the understanding of how protein complexes interact to form complex supra-molecular structures, but is also of great significance to medicine, as many mutations in genes encoding sarcomeric proteins cause muscle diseases such as congenital myopathy and dilated cardiac hypertrophy [1], [2].

The striated appearance of myofibrils is readily detectable under the polarized light microscope as alternating light and dark bands corresponding to I-bands and A-bands, respectively (Figure 1A). Thin filaments are built of actins as well as their associated tropomyosin (Tm) and troponin (Tn) complex (troponin T/TnT, troponin I/TnI and troponin C/TnC) proteins, and are anchored at the Z-line that demarcates the middle of the I-bands. The Z-line itself contains various structural proteins such as α-actinin, titin and Zasp. Thin filaments and Z lines are connected with each other and are often referred to as the “I-Z-I” complex. Thick filaments on the other hand are attached to the M-line situated at the center of A-bands and are composed of muscle myosin represented by two myosin heavy chains (MHCs) and four muscle light chains (MLCs).

A number of models have been put forward to explain how sarcomeric proteins are assembled into a highly ordered structure. One model proposes that I-Z-I complexes and bipolar myosin filaments assemble independently before joining [3]. The main observations in support of this view are that free-floating A band structures can be detected in the absence of actin, and that irregular Z-lines can be detected with attached thin filaments in muscles that lack myosin [3]–[5]. A second model, supported by the antibody staining of chicken cardiomyocytes fixed at different times after spreading in culture [6], proposes that premyofibrils, characterized by interdigitating banded patterns of I-Z-I complexes and non-muscle myosins, form *de novo* near the cell membrane and develop into mature myofibrils. This model proposes that a replacement of the non-muscle myosin filaments with muscle myosin filaments occurs during the transition from premyofibril to myofibril and that discrete aggregates of Z bodies along premyofibrils grow and fuse laterally into Z lines [6], [7]. A third model emphasizes the scaffolding role of the giant protein titin during myofibrillogenesis. According to this model, the N-terminal domain of titin interacts with the I-Z-I complex and the subsequent unfolded C-terminal region associates with the M-line, thus driving the interdigitation of the I-Z-I complex and myosin. In support of this model, using titin specific epitopes that distinguish between the N or C region, the M-band periodic pattern of titin is detected later than its Z-line pattern [8], [9]. Finally, a fourth model emphasizes the role of integrin adhesion complex in sarcomere assembly as starting sites of actin/thin filaments polymerization [10]. This model is supported by genetic studies in *C. elegans*, *Drosophila* and mice showing that integrin signaling pathway components are required for sarcomere assembly [11]–[15].

Although not necessarily mutually exclusive, these models are based on the analysis of sarcomeric structures in either wild-type or mutant backgrounds using electron microscopy and immuno-histochemistry approaches, and have been limited by the lack of a number of antibodies against key sarcomeric proteins. Thus, we decided to reexamine this process using new reagents and approaches to systematically investigate the myofibril assembly process. In particular, we used RNAi in a primary muscle cell culture assay to systematically explore the functional relationships among sarcomeric proteins, combined with detailed in vivo analysis of available muscle mutants. Previously, we have shown that primary muscle cells can be used to study sarcomere organization using RNAi [16], [17]. For example, we identified from an RNAi screen in primary muscle cells the *sarcomere length short* (*sals*) gene and showed that it is required for thin filament lengthening both in culture and *in vivo* [18].

Here, using antibody staining of sarcomeric proteins in mutant animals and cultured primary muscle cells, we document how sarcomere formation is a highly coordinated process mediated by the assembly of multiple latent functional complexes. We find that I-Z-I proteins and myosin filaments are two independent complexes that interact with each other to provide the alignment feature of the myofibrils, thus supporting the “independent” model described above [3]. In addition, we found that this interdigitation is at least mediated by the troponins-tropomyosin (Tns-Tm) complex that is critical for sarcomere contraction. Further, we show that the newly identified zipper/Zasp/α-actinin complex functions as a tension sensor to stabilize the I-Z-I complex. Finally, we find that integrin is essential for the interdigitation of thin and thick filaments, but is not required for actin/thin filament assembly as previously proposed. Altogether, our studies indicate that there are no intermediate steps for sarcomere assembly and that disruption of any protein complex leads to loss of muscle striation, thus leading to the proposal that sarcomere formation occurs by the coordinated assembly of multiple latent protein complexes.

## Results/Discussion

### Roles of MHC and non-muscle myosin in sarcomere formation

Previous studies have established that the first sarcomeric components to appear are the actin filaments that assemble into I-Z-I complexes [8], [19], [20]. This step is followed by the infiltration of muscle myosin into nascent myofibrils, a process that is not well understood and that may be mediated by non-muscle myosin [3], [6], [8], [21].

To clarify the process by which I-Z-I and the myosin filaments assemble, we analyzed the striation pattern of sarcomeres in *Mhc^1^* mutants that lack thick filaments. *Mhc^1^* contains a 101 base-pair (bp) deletion in the *Mhc* gene resulting in truncated MHC proteins lacking most of rod-like sub-fragment 2 that contributes to the backbone of the thick filament. Previous electron microscopic studies have shown that mutant muscles for the *Mhc^1^* amorphic allele completely lack any discernable thick filaments [22]. Wild-type and *Mhc^1^* mutant embryos were dissected at stage 17, when sarcomeres begin to assemble and can be detected in the wild-type body wall muscles [23] (see Methods). Muscles were then stained with various antibodies against sarcomeric proteins. While wild-type muscles displayed well-defined striated myofibrils when visualized with phalloidin staining and anti-α-actinin antibodies, highlighting actin filaments and Z-lines respectively (upper panels in Figure 1B), homozygous *Mhc^1^* mutant muscles no longer showed localized Z-lines (bottom panels in Figure 1B). This observation is consistent with the phenotypes of *Mhc* deficient primary muscles that are derived either from wild-type cells treated with *Mhc* dsRNAs or from cells dissociated from *Mhc^1^* mutant embryos (Figure S1A and S1B). Altogether, these results indicate that I-Z-I proteins cannot align into striation in the absence of MHC.

Next, to test the role of non-muscle myosin in sarcomere formation, we stained *Mhc^1^* mutant muscles with antibodies against zipper that corresponds to the only *Drosophila* non-muscle myosin. One prediction of the model emphasizing the role of non-muscle myosin is that removal of MHC would only have subtle effects on the zipper sarcomeric striation pattern, as non-muscle myosin would be involved in the formation of premyofibrils. We find that zipper is strongly localized to Z lines but not to thick filaments (upper panels in Figure 1C), and that removal of MHC resulted in no obvious zipper periodic pattern (bottom panels in Figure 1C). These results suggest that, at least in *Drosophila*, intermediate non-muscle myosin-containing premyofibrils do not exist during myofibril assembly. Further, examination of other sarcomeric components (β-integrin (Figure 1B), Mlp84B, titin, and Zasp (data not shown)) indicates that elimination of MHC leads to a complete disruption of the distribution of these proteins in myofibrils.

As MHC is expressed in embryonic muscles several hours prior to sarcomere organization, one possibility is that MHC is required for the organization of proteins at later stages. To exclude this possibility, we stained embryos at stage 15–16 when many sarcomere proteins become enriched at muscle attachment sites. Removal of MHC had no effect on the distribution of integrin, α-actinin, zipper, kettin, Zasp and Mlp84B at the attachment sites (Figures S2, S3, S4), strongly suggesting that formation of muscle attachments is a MHC-independent process.

To investigate the role of MHC in sarcomeric organization at later stages following sarcomere formation, we knocked down *Mhc* in third instar larvae by RNAi (*Mhc-Gal4;UAS-Mhc-hp*) and examined the distribution of sarcomeric components. Compared to control animals (*Mhc-Gal4/+*) the degree of loss of muscle sarcomere striation correlated well with the reduction in the MHC expression level, as revealed by the fluorescent staining for α-actinin, Zasp and MHC (Figure S5). Altogether, these results indicate a critical role for MHC not only in sarcomere formation but also in its maintenance.

### Function of the troponins-tropomyosin complex

**Figure 2:**
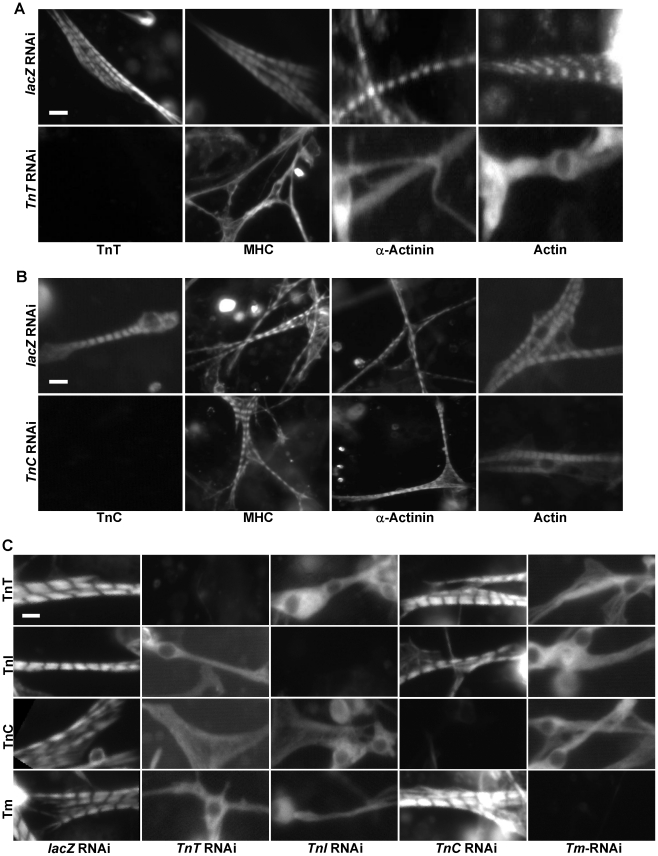
Tns-Tm complexes play an important role in sarcomere assembly. (A,B) Primary muscle cells were isolated from *Oregon R* embryos and treated with dsRNA against *lacZ* (A and B, upper panels), *TnT* (A. lower panels), or *TnC* (B. lower panels). Sarcomeric organization was evaluated following staining using polyclonal antibodies against MHC, α-actinin or actin. The efficacy of *TnT* and *TnC* RNAi knock-downs were evaluated using anti-TnT and anti-TnC specific antibodies, respectively. Note that while removal of TnT has severe effects on the striated organization of the sarcomere, depletion of TnC has little effect. (C) To determine the relationships between TnT, TnI, TnC and Tm, primary muscle cells were treated with *TnT, TnI, TnC* or *Tm* RNAi and stained with antibodies against TnT, TnI, TnC and Tm, respectively. Note the severe effects on the striated pattern in the absence of TnT, TnI and Tm, but not TnC. Scale bars: 10 µm.

The Tns-Tm (TnT/TnI/TnC/Tm) complex has a well-established role in acto-myosin interactions in response to changes in Ca^2+^ level. Since MHC is required for sarcomere formation, we analyzed whether this complex also participates in this process. Because complete null mutations in these genes are not available, and primary muscle RNAi has been successfully used to characterize sarcomere assembly [18], we used primary muscle cell cultures to assess the roles of Tns in sarcomerogenesis. Primary muscles were prepared from wild-type embryos, treated with various dsRNAs targeting different Tns, and stained with the three major sarcomeric proteins actin, α-actinin, and MHC. Depletion of *TnT* or *TnI* eliminated the striation pattern, indicating that both are essential for sarcomere formation (Figure 2A and Figure S6A). This is consistent with the *in vivo* sarcomeric disassembly phenotypes associated with loss-of-function *TnT* mutations described in mice and zebrafish and of *TnI* in the indirect *Drosophila* flight muscle [24]–[26]. As previously proposed by Sparrow and colleagues [25], since TnI inhibits the generation of acto-myosin force for muscle contraction in the absence of Ca^2+^ and TnT is involved in the attachment of the Tn complex to the thin filament, the defects observed in the absence of either TnT or TnI are likely caused by the upregulation of acto-myosin interactions between thin and thick filaments as they assemble.

Interestingly, we expected that knock-down of the Ca^2+^-binding protein TnC would affect sarcomere organization as loss of *TnC* should desensitize the response to Ca^2+^ concentration and reduce acto-myosin interaction between thin and thick filaments. However, knock-down of *TnC* did not lead to any significant change in MHC/actin/α-actinin striation (Figure 2B). This non-essential role of TnC in sarcomere assembly underscores the importance of direct interactions between Tns and thin filament in sarcomere formation since, unlike TnT and TnI, TnC does not directly interact with actin and Tm [27].

Since Tns always function together with Tm to mediate actin-myosin interactions, we next examined the role of Tm in sarcomere organization. Staining of primary muscle cells treated with *Tm* dsRNAs with anti-actin, anti-α-actinin and anti-MHC demonstrated that Tm is another critical component participating in sarcomere assembly (Figure S6B). As TnT, TnI and Tm are all essential for sarcomere formation, we tested whether these three proteins are assembled in a sequential manner into sarcomeres, as observed in the case of other protein complexes involved in organelle assembly [28]. Thus, we depleted systematically each component of the Tns-Tm complex by RNAi and examined the overall organization of sarcomeres with antibodies against Tns and Tm. Strikingly, removal of *TnT*, *TnI* or *Tm* led to random distribution of all remaining proteins, indicating that there is no loading sequence for Tns-Tm complex during sarcomere assembly (Figure 2C).

To test the possibility that removal of the Tns-Tm complex would lead to up-regulation of acto-myosin contraction by exposing the MHC binding sites on actin, and as a result causes disruption of the myofibril structure, we performed a time-course analysis of sarcomere striation. Typically, a clear striation pattern indicated by actin staining can be first observed in wild-type primary muscle cells culture at 3 days after plating at 25°C ([16], Figure S6C). In *TnT* RNAi treated cells, no striation could be detected at that time-point or later (Figure S6C) suggesting that the Tns-Tm complex plays an important role in the initial sarcomere assembly by allowing stable interdigitation of thin and thick filaments. Similar results were also obtained from *TnI* or *Tm* RNAi treated primary muscle cells (data not shown). Interestingly, we did not observe a sequence of events in the recruitment of Tns and Tm, suggesting that the Tns-Tm complex is recruited as a complex to the sarcomere.

### Relationship between MHC and I-Z-I

I-Z-I complexes are composed of thin filaments associated with nascent Z-lines and are the first identifiable structures during early myofibrillogenesis [3]. Subsequently, MHC has been hypothesized to assemble into thick filaments and to interdigitate with thin filaments to form sarcomeres.

**Figure 3:**
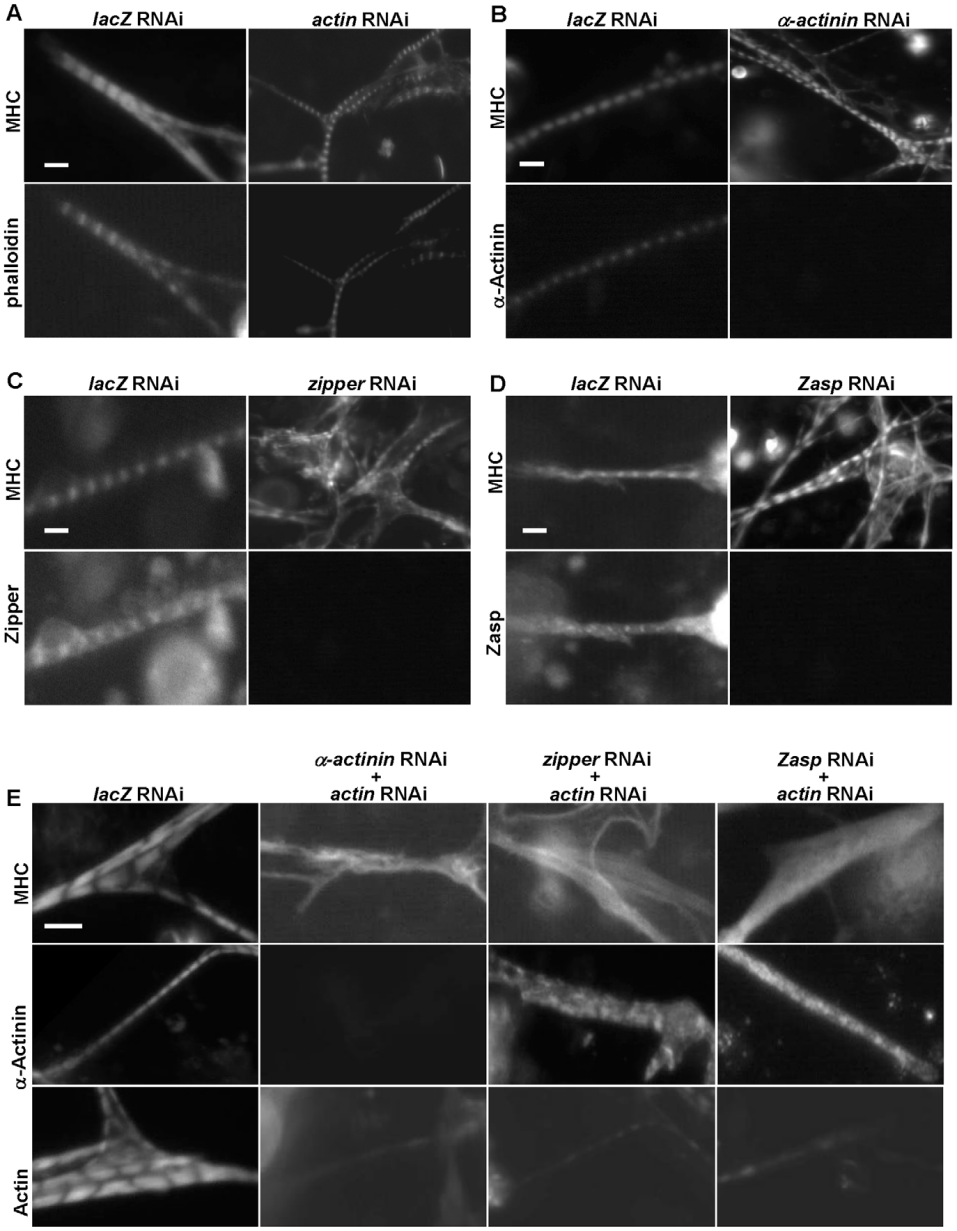
The I-Z-I complex is required for sarcomere organization. (A–D) DsRNAs against *actin* (A), *α–actinin* (B), *zipper* (C), *Zasp* (D) or control *lacZ* were applied to primary muscle cell cultures. Phalloidin, anti-α-actinin, anti-zipper and anti-Zasp antibodies revealed the knock-down efficiencies of the various dsRNAs. The structure of the muscles was monitored following staining using an anti-MHC antibody. Note that in the absence of a single component of I-Z-I, thick filament remains in a striated pattern. As a control, we treated muscle cells with *actin* RNAi in combination with *lacZ* RNAi or *mlp84B* RNAi, which did not produce any significant effects on muscle striation pattern (data not shown). (E) DsRNAs against α-*actinin, zipper* and *Zasp* were individually mixed with a dsRNA against *actin* in a 1∶1 ratio. The sarcomeric organization of primary muscle cells was analyzed following staining using anti-MHC, anti-α-actinin and anti-actin antibodies, respectively. Note the absence of MHC striation when two I-Z-I components are depleted. We also confirmed the localization pattern of Z-line proteins with our available antibodies after double knock-down and showed absence of striation (data not shown), indicating that the I-Z-I complex is critical for the periodic localization of other functional complexes such as MHC and Tns-Tm. Scale bars: 10 µm.

To test directly this model, we knocked down each component of the I-Z-I complex including *actin*, *zipper*, *Zasp*, *α-actinin* and *Mlp84B* in primary muscle cells [29]–[31]. First, when *actin* was depleted by RNAi, primary muscles stained with anti-MHC showed much shorter and thinner striated myofibrils (Figure 3A). Since actin is essential for cell survival, the presence of striated myofibrils suggests that residual actin remains, which is consistent with the weak phalloidin staining observed (Figure 3A). We attempted to increase the severity of the RNAi phenotype by using a greater amount of dsRNAs (Figures S7 and S8), however, thin and shortened actin filaments were still present. There are six actin isoforms (Act5C, Act42A, Act57B, Act79B, Act87E and Act88F) in *Drosophila*, which only differ in a few amino acids. Act5C, Act42A, Act57B, Act87E are believed to be expressed in larval body wall muscles [32]. Act5C and Act42A are cytoplasmic actins uniformly expressed in all cell types, including muscles, whereas Act57B and Act87E are muscle specific. Although the dsRNAs we used target all actin isoforms, our primary cell RNAi protocol may not allow complete depletion of actin isoforms, such as *Act42A* and *Act5C*, as they are expressed in myoblasts [17]. Since, it is possible that the initial sarcomere assembly in muscles utilizes cytoplasmic actins as building blocks, we speculate that the shortened actin filaments (Figure 3A) results from residual amount of cytoplasmic Act5C and Act42. In further support of this model, it has been previously shown that in vertebrate muscles, the actin turn-over rate in thin filaments is over 100 fold lower than that in cytoplasmic actin filaments [33]. Thus, the extreme slow turn-over rate of these cytoplasmic actins, initially incorporated into thin filaments, could be sufficient to allow the formation of short sarcomeres [18].

Second, we investigated the role of Z-line components, such as α-actinin, zipper, Zasp, and Mlp84B, in sarcomere organization (Figure 3B–3D and data not shown). No significant changes in the striation pattern, as monitored by MHC staining, were observed in the *α-actinin* knock-down, indicating that α-actinin is not required for thick filament organization ([34], Figure 3B). Knock-down of *zipper* led to fuzzy but discernable striation (Figure 3C and Figure S9A, S9B). Silencing of *Zasp* gave similar results as seen from *zipper* RNAi knock-down results (Figure 3D). Further, inactivation of *Mlp84B* appeared to have a negligible effect on the muscle sarcomere structure as determined by anti-MHC staining (data not shown). Finally, previous electron microscopic analysis of *α-actinin, zasp, and Mlp84B* mutant muscles have shown that Z-disc structures are still present in these muscles [30], [31], [34], indicating that knock-down of these sarcomeric components does not affect the initial formation of sarcomeres.

Because removal of I-Z-I components individually did not lead to dramatic MHC disorganization, we tested whether these proteins act in a partially redundant manner by performing combinatorial perturbations. Simultaneous knock-downs of *actin* and one of Z-line proteins such as *α-actinin, zipper or Zasp* were associated with complete loss of striation and “stress fiber-like” MHC phenotypes reminiscent of randomized MHC filaments (Figure 3E). These results suggest that myosin can assemble into thick filaments independently of I-Z-I complexes but that the sarcomeric structure of MHC filaments require the ordered arranged I-Z-I complexes and vice versa.

### Zipper/Zasp/α-actinin may act as a tension sensor

**Figure 4:**
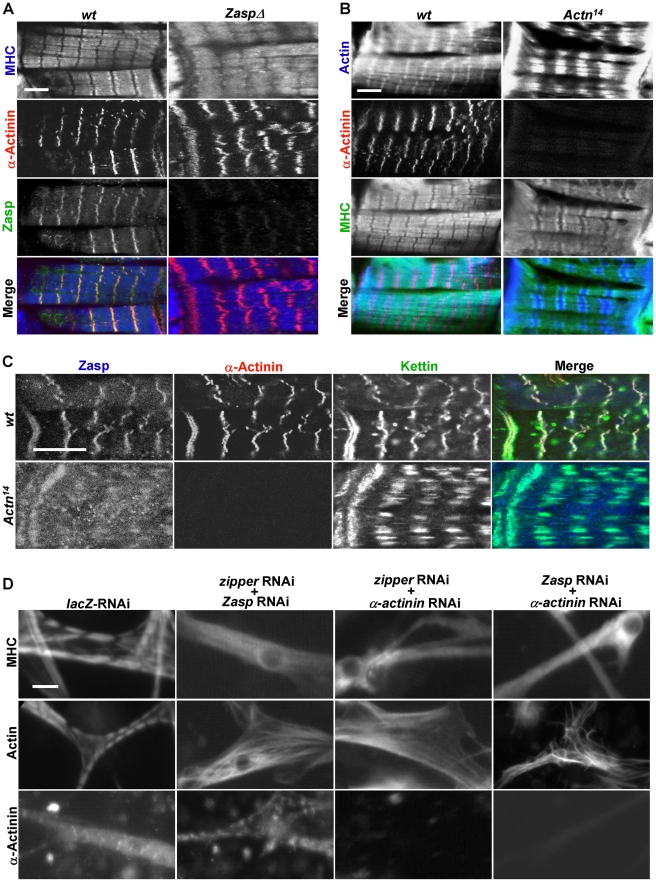
Zipper/Zasp/α-actinin acts as a tension sensor to regulate sarcomere assembly. (A) Confocal micrographs of control muscles of a stage 17 wild-type embryo (left panels) and age-comparable *zasp* null mutant muscles (right panels) stained for MHC (blue in merge), α-actinin (red in merge) and Zasp (green in merge). Scale bar: 10 µm. (B) Confocal micrographs of control muscles of a stage 17 wild-type embryo (left panels) and same stage *α-actinin* null mutant muscles from *Actn^14^* (right panels) stained for actin (blue in merge), α-actinin (red in merge) and MHC (green in merge). Scale bar: 10 µm. (C) Fluorescent confocal micrographs of control muscles of a stage 17 wild-type embryo (top panels) and *α-actinin* null mutant muscles from *Actn^14^* of same stage (bottom panels) stained for Zasp (blue in merge), α-actinin (red in merge) and kettin (green in merge). Note that *α-actinin* null mutant muscles still have striated sarcomeres, but with expanded Z lines. Scale bar: 10 µm. (D) Primary muscle cells were treated with combinations of dsRNAs targeting components of the zipper/Zasp/α-actinin complex. Muscle striation was evaluated using anti-MHC, anti-actin and anti-α-actinin antibodies. Scale bar: 10 µm.

Previous studies have reported that *zasp* and *α-actinin* mutant muscles exhibit some defects in sarcomeric structures [30], [34], which contrasts with our RNAi results that Zasp is not required for initial sarcomere assembly. To rule out the possibility that our results do not simply reflect a partial knock-down due to RNAi, we examined the muscle phenotypes of *zasp* and *α-actinin* amorphic mutants at the stage when myofibrils just start to form and display striated bands for MHC, α-actinin and Zasp (left panels in Figure 4A). At that stage, we could detect regularly spaced staining distribution of these proteins indicating that Zasp is not required for initial sarcomere assembly (Figure 4A). At later stages however, striation in *zasp* mutant embryos began to disappear, suggesting that Zasp is required for sarcomere maintenance (data not shown) [30].

Next, we analyzed *α-actinin* mutants using anti-actin and anti-MHC antibodies and found that their Z lines were expanded, a phenotype consistent with previous electron microscopy studies (Figure 4B) [34]. Expanded Z lines were also present in *α-actinin* mutant muscles stained with kettin and Zasp antibodies (Figure 4C), indicating that α-actinin is required for Z-line condensation but not for sarcomere assembly or recruitment of other Z line proteins such as titin/kettin and Zasp. In contrast, in *kettin* mutant muscles, no striation pattern was detected for any markers [35], [36]. Collectively, these observations point out that the recruitment of Zasp, kettin and α-actinin to the Z-line does not occur in a step-wise fashion. Instead, it is more likely that they cooperate with each other in stabilizing Z-lines and are recruited concomitantly. Notably, zipper/Zasp/α-actinin are highly enriched at the muscle attachment sites and act downstream of integrin to link the muscle cytoskeleton to the cell membrane, suggesting that zipper/Zasp/α-actinin may act as a tension sensor to mediate the interaction between integrin and the internal cytoskeleton. Interestingly, single depletion of *zipper*, *Zasp* or *α-actinin* led to less severe phenotypes in sarcomere assembly in primary muscle cell cultures than *in vivo* (Figure 3A–3D), most likely reflecting the weaker stress on fibers *in vitro* versus *in vivo*.

To test this idea, we induced muscle stress in primary culture by increasing muscle cell intracellular calcium (Ca^2+^) concentration. To increase contraction by augmenting the level of intracellular Ca^2+^, we knocked-down the plasma membrane calcium ATPase (PMCA) that pumps intracellular Ca^2+^ from the inside of cells [18]. We first found conditions where primary muscles were able to maintain relatively normal sarcomere structures when treated with 40 ng of dsRNAs against *PMCA* (Figure S10A). We next used this sensitized condition as a stress background for primary muscles in culture to examine the effect of reduction of those tension sensor components by double RNAi knock-down. Consistent with the idea that zipper/Zasp/α-actinin may act as a tension sensor, double knock-down with the mixture of dsRNAs targeting *PMCA* and either *zipper* or *Zasp*, or *α-actinin* led to significant disruption of sarcomeric organization, although primary muscles treated with either 40 ng of dsRNAs targeting *PMCA*, or 250 ng of dsRNAs targeting *zipper* or *Zasp*, or *α-actinin* still had normal sarcomere structures (Figure S10B). We further looked at the distribution of these tension-sensor proteins in muscles from paralyzed embryos caused by the mutation in *immaculate connections* (*imac*), a gene that encodes a neuron-specific kinesin required for presynaptic maturation [37]. In agreement with our previous conclusions, these tension-sensor components were still present at muscle ends, although the sarcomere structures were disrupted, presumably due to less tension on the muscles in these animals. (Figure S11).

We then explored the functional interaction among zipper, Zasp or α-actinin, given that single knock-down of these Z-line proteins did not reveal a requirement in sarcomere assembly. Strikingly, removal of *zipper, Zasp*, and *α-actinin* together led to a complete loss of striation (Figure 4D), suggesting that they function redundantly in sarcomere assembly. Thus, single knock-down of only one of these tension sensor proteins is not sufficient to disrupt the whole complex, however, the combined removal of these components leads to a collapse of the entire sarcomere.

Finally, we examined the role of titin/kettin in sarcomere formation. Titin is a large protein localized at Z-line that contains multiple domains important for interactions among I-Z-I components. It has been proposed to act as a third filament to regulate plasticity of sarcomere. Consistent with its predominant role in the sarcomere, knock-down of *titin* is associated with complete disorganization of the sarcomere both *in vitro* and *in vivo* (Figure S12A and S12B). Removal of the scaffold protein titin may be equivalent to simultaneously disturbing the association of I-Z-I proteins and mimics the combinational knock-down of components from I-Z-I complex.

### Role of integrin in sarcomere assembly

Genetic studies in *C. elegans*, *Drosophila* and mice have implicated the integrin pathway in sarcomere assembly [11]–[14]. Although, disruption of integrins is associated with a complete loss of myofibril striation in muscle cells [14], the exact role of integrins in sarcomere assembly has not been explored. Recently, integrin has been proposed to pave the way for sarcomere assembly by initiating the assembly of actin filaments at the muscle cell membrane [10]. A prediction of this model is that, in the absence of myosin, I-Z-I complexes should be tethered to costameres, which link muscle cell membrane to myofibrils, with integrins. To test this model, we dissected embryos from wild-type and *Mhc^1^* mutant embryos that lack MHC and stained them with antibodies against integrin, actin and α-actinin. In wild-type muscles, β-integrin is highly concentrated at the muscle attachment sites and relatively weakly stained at the position of the costameres with a striated pattern that appears to align with Z lines (Figure 1B). In contrast, we could not detect any striation for β-integrin at the location of the costameres in *Mhc^1^* mutant muscles. No striation could be detected for actin and α-actinin either. However, the pattern stained by actin and α-actinin did not match that of β-integrin on the membrane, indicating that there is no obvious connection between Z lines and β-integrin in *Mhc^1^* mutant muscles. These results suggest that integrins do not appear to be involved in the assembly of I-Z-I complexes, and that MHC is required for integrins to be aligned with Z-lines.

**Figure 5:**
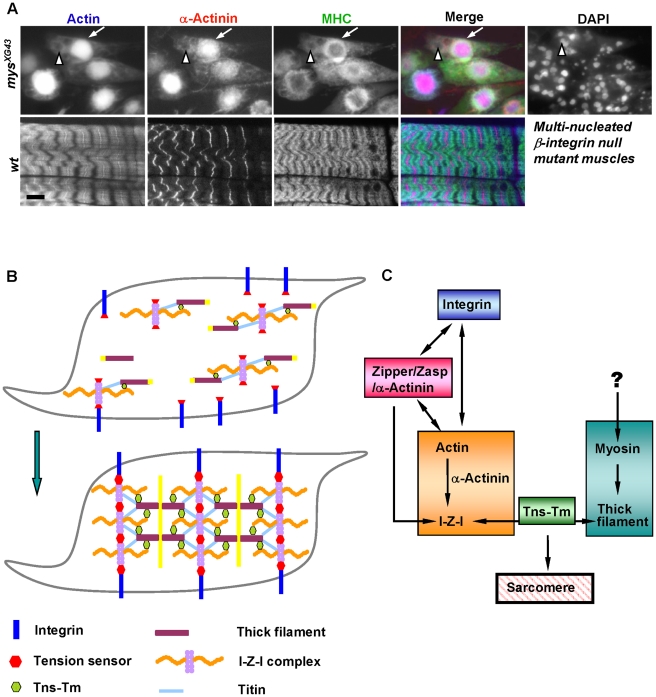
Integrin is essential for sarcomere assembly and model. (A) Fluorescent confocal micrographs of stage 17 wild-type muscles (left panels) and *mys* null mutant muscles of the same age (right panels) stained for actin (blue in merge), α-actinin (red in merge) and MHC (green in merge). DAPI staining reveals the multinucleated *mys* null mutant muscles. Note that I-Z-I collapsed to the center of muscles in the *mys* mutant, where thick filaments were mostly excluded. Scale bar: 10 µm. (B) A “two-state sarcomere assembly” model. Prior to sarcomere formation, various complexes, including integrin, tension sensor, I-Z-I complex, MHC filament and Tns-Tm, are assembled independently. Subsequently, the various complexes assemble and interact with the integrin pathway responsible for sarcomere stretching. Removal of any one of these complexes leads to a collapse and disorganization of the entire system. (C) Relationships between the sarcomeric functional complexes. The arrows indicate the interaction among these complexes as determined by the results presented in this study.

We further investigated the expression patterns of muscle markers in both wild-type and *integrin* null mutant (*mys^XG43^*) muscles of stage 17 embryos when myofibrils start to form [30]. At that stage, β-integrin deficient muscles contained multiple nuclei, indicating that the myoblast fusion process took place normally, and were not yet completely rounded up (Figure 5A). Further, in agreement with a previous report [14], sarcomeric structures in these muscles were totally disrupted. Immunostaining using antibodies against actin, MHC and α-actinin revealed that the majority of both actin filaments and Z lines collapsed to the center of muscle cells and formed aggregates where myosin filaments were excluded (Figure 5A). This result shows that association of actin and α-actinin occurs even in the absence of integrins, implying that I-Z-I complex formation may not require integrins. Furthermore, in the absence of integrins, thin and thick filaments cannot interdigitate with each other to form sarcomeres. Altogether, our immunohistochemical study results are consistent with previous electron microscopy analysis of integrin mutant muscles, which contained disorganized thin and thick filaments distributed in the central region of myotubes [38]. Further, a recent report has elegantly dissected the dual role of integrin in muscle attachment and sarcomeric organization using an inducible targeted RNAi system [39]. This study revealed that reduction of integrin led to the dissociation of Z-disc proteins into small dots, whereas muscle attachment sites were not affected, and thus suggested a role for integrin in maintainance of sarcomere structures that may be different from its role at attachment sites. Altogether, based on the data from our study and others [14], [38], integrins appear to play very important roles in maintaining the tension at the cell periphery for organization of sarcomere structures. We propose that integrins serve as anchor points for the floating I-Z-I complex and provide tensions that allow the interdigitation of thin and thick filaments.

### A model for sarcomere assembly

Based on our *in vitro* and *in viv*o mutant analyses, we propose that sarcomeric proteins exist in two states: one being the chaotic but independently assembled differential functional complexes, and the other one being the highly ordered suprastructure made from these complexes (Figure 5B). We propose that several functional complexes including integrin, zipper/Zasp/α-actinin, I-Z-I, Tns-Tm and MHC first assemble independently (as described in shadowed boxes in Figure 5C). Subsequently, we propose that integrins serve as anchor points for the floating I-Z-I complex starting from muscle ends, and provide tensions that allow the interdigitation of thin and thick filaments for de novo sarcomere assembly. This in turn may facilitate the spatial organization of integrins on cell membrane by linking the integrins to the underlying cytoskeleton and “towing” the integrin adhesion complex into periodic position, which may further strengthen newly formed myofibril structures. In the context of our loss of function studies, only those perturbations that affect the formation of the independent complexes will lead to the failure in assembly of striated sarcomeres. If depletion of any one protein has negligible effects on the localization of the complex where it belongs to, then sarcomere striation will be maintained. Altogether, our study provides a comprehensive analysis of the functional effects of perturbation of sarcomeric proteins on myofibril assembly. Because of the similarities between *Drosophila* and vertebrate sarcomeres the “wo-state”assembly model that we have proposed should prove relevant to striated muscles.

## Methods

### *Drosophila* genetics

*Oregon R* was used as a wild-type strain. Mutations in genes encoding sarcomeric proteins are: *Mhc^1^*, *zasp^Δ^*, *mys^XG43^*, *Actn^14^*, *imac^170^* ([11], [22], [30], [34]), and *zip^p1215^*. Mutant strains were balanced with *twi-GAL4>UAS-GFP* or *Kr-GFP* and mutant primary muscle cells were identified by lack of GFP expression. For RNAi experiments, *Mhc-Gal4* [40] and *UAS-Mhc hp* [41] flies were used.

### Embryonic primary cell culture

Embryonic primary cell cultures were established as previously described [17], [42]. Eggs were collected on molasses plates for 2 hrs and incubated for 4 hrs at 25°C. Embryos were dechorionated in 50% bleach for 3 mins, rinsed thoroughly with 70% ethanol and sterilized water, and dissociated into a cell suspension using Dounce homogenizers (VWR Scientific, Seattle, WA) in Shields and Sang M3 medium (Sigma). Cell suspensions were spun once at 40 g for 10 mins to pellet tissue debris, large cell clumps and vitelline membranes. Supernatants were then transferred to a fresh tube and spun at 360 g for 10 mins to pellet the cells. Cells were washed once and re-suspended in primary cell medium (10% heat-inactivated fetal bovine serum from JRH Biosciences, 10 mU/ml bovine insulin in M3 medium from Sigma). Cells were seeded and grown in 384-well optically clear plastic plates (Costar) at 1.7–2.5×10^5^ cells/cm^2^.

### Primary cell RNAi treatment

DsRNAs synthesis and RNAi treatment were performed according to the DRSC protocols (http://flyRNAi.org) using amplicons to target various genes: DRSC23472 and DRSC23961 for *actin*; DRSC14106 for α-*actinin*; DRSC03367 and DRSC25959 for *Mhc*; DRSC28798 for *troponin T*; DRSC29330 for *troponin I*; DRSC04916 and DRSC07564 and DRSC11307 for *troponin C*; DRSC16887 and DRSC26884 for *tropomyosin*; DRSC04725 and DRSC22489 and DRSC28416 for *zipper*; DRSC07121 for *Zasp*; DRSC20341 and DRSC39458 for *integrin*; DRSC34373 and DRSC34374 for *PMCA*. DsRNA against *lacZ* was used as a control in all the above experiments. Primary muscle cells were prepared as described above and seeded at approximately 4×10^4^ cells per well in plates containing dsRNAs. After 22 hrs in serum-free M3 medium, additional serum-containing culture medium was added to bring the solution to a final concentration of 10% FCS. For multiple knock-down experiments, dsRNAs were mixed into the plate before adding the muscle cells.

### Immunofluorescence microscopy

Flies were allowed to lay eggs for 2 hrs and embryos further developed at 25°C for 14–16 hrs to reach stage 17 of embryogenesis. The body wall muscles of staged embryos were dissected by following methods described previously [16]. Dissected muscles or primary cells were fixed with 4% formaldehyde. Cells were stained overnight at 4°C with the first antibody or phalloidin Alexa Fluor 568 (Molecular Probes; 1∶2000) and DAPI (Sigma, 1∶5000) in PBTB (PBS, 0.1% Triton X-100, 1% BSA), washed once in PBS and left in PBS containing 0.02% NaN_3_. For whole mount embryo stainings at stages 15–16, standard protocol was followed, including dechorionization, fixation, rehydration, and staining with antibodies.

Antibodies used in this study: rat anti-actin, anti-MHC, anti-TnT, anti-TnC, anti-Tm (1∶500) (The Babraham Institute, Cambridge, UK), mouse anti-α-actinin (1∶100) (from Dr J. Saide, Boston University, Boston, MA), rabbit anti-zipper (1∶500) (from Dr. D. Kiehart), rabbit anti-Zasp (1∶1000) (from Dr. F. Schöck), anti-kettin (1∶1000) (Dr. D. Andrew), anti-PS3/anti-β-integrin (1∶100) (Developmental studies hybridoma bank). The second antibodies from invitrogen include Alexa 488 and Alexa 594.

### Generation of anti-TnI antibody

Full-length TnI was cloned into pGEX4T-1 and GST fusion protein was purified according to the protocols described from Pharmacia. The recombinant protein was sent to Abmart Antibody Company (Shanghai, China) and the antibody was purified using the original antigen. TnI antibody specificity was confirmed by cell staining results of *TnT* RNAi-treated muscle cells.

### Real-time PCR

qRT-PCR was performed to analyze the RNA level of actin in *Drosophila* S2 cells treated by dsRNA against *lacZ* or *actin* at different concentrations followed by reverse transcription using the SuperScript first-strand synthesis kit (Invitrogen). The experiment was carried out in Stratagene MX4000 thermocycler using SyBy GreenTM detection protocol. Sequencing primers for *Drosophila* actin, forward: GAGCGCGGTTACAGCTTCA, reverse: TCCTTGATGTCGCGCACA.

## Supporting Information

Figure S1 MHC is required for sarcomere formation. (A,B) Primary muscle culture cells were isolated from *Mhc-GFP* and treated with *Mhc* dsRNA. (A) *Mhc^1^* mutant embryos. (B) Anti-MHC staining was used to assess the knock-down efficiency or to identify *Mhc* mutant primary muscle cells. Cultures were immunostained using anti-actin or anti-α-actinin to analyze sarcomeric structures. Anti-zipper antibody was used to analyze the localization of zipper in the absence of MHC. Scale bars: 10 µm.
(2.20 MB TIF)

Figure S2 Removal of MHC has no effect on the localization of integrin and α-actinin. (A,B) Staining of integrin and α-actinin was performed in both wild-type and *Mhc^1^* mutant stage 15–16 embryos. MHC antibody was used to analyze *Mhc^1^* null allele. Both MHC and other sarcomeric protein stainings were merged to check their localization relationships. Scale bar: 20 µm.
(7.76 MB TIF)

Figure S3 Localization of zipper and kettin at muscle attachment sites in the absence of MHC. (A,B) Zipper and kettin were stained with antibodies to show their muscle attachment site localization in the presence and absence of MHC in stage 15–16 embryos. Scale bar: 20 µm.
(6.09 MB TIF)

Figure S4 Zasp and Mlp84B localize at muscle attachment site in a MHC-independent manner. (A,B) Zasp and Mlp84B distribution were assessed by their antibody stainings and merged with MHC staining to check their localization relationships in 15–16 stage embryos. Scale bars: 20 µm.
(6.41 MB TIF)

Figure S5 Localization of sarcomeric components in larval muscles is disrupted upon MHC reduction. Confocal micrographs of second instar larval body wall muscles from control animal (top panels) and age comparable muscles from a larva carrying transgenes of *Mhc-Gal4;UAS-Mhc hp* (bottom panels) stained for MHC (green in merge), α-actinin (blue in merge) and Zasp (red in merge). Scale bar: 50 µm. Note that the presence of striated organization of these sarcomeric components correlated well with the presence of MHC expression (arrowheads at bottom panels), while loss of MHC expression led to disruption of sarcomere striation and distribution of these sarcomeric proteins.
(2.73 MB TIF)

Figure S6 The Tn-Tm complex is essential for sarcomere assembly. (A,B) DsRNAs against *TnI* or *Tm* were applied to primary muscle cells, and anti-TnI and anti-Tm antibodies were used to document the knock-down effectiveness. The sarcomeric organization of treated muscles was analyzed using anti-actin and anti-α-actinin antibodies. (C) *TnT* knock-down time course experiment. No striation was observed in *TnT* RNAi-treated primary muscle cells, even at 3 days after plating when the sarcomeres begin to form in the *lacZ* RNAi control. Muscle cultures were stained with anti-MHC antibody. Scale bars: 10 µm.
(3.84 MB TIF)

Figure S7 Persistent arrest of residual actin protein in myofibril after dsRNA treatment. Different amounts of *actin* dsRNA were added to primary muscle cell culture from 50 ng to 1 mg. *lacZ* dsRNA was used as a control. Anti-actin antibody was applied for analysis of residual actin signal and anti-MHC for muscle structure. Scale bars: 10 µm.
(3.38 MB TIF)

Figure S8 Quantitative RT-PCR analysis of *actin* RNAi efficiency. 250 ng or 800 ng of dsRNA against *actin* were applied to Drosophila S2 cells in comparison of treatment with 250 ng of *lacZ* dsRNA. Quantitative RT-PCR analysis was performed to assess the actin knock-down effectiveness. The amount of actin mRNA from *lacZ* dsRNA treatment was used as a normalization control.
(0.27 MB TIF)

Figure S9 Zipper is not exclusively required for sarcomere striation. (A) Primary muscle cells treated with *zipper* dsRNA and stained using anti-MHC, anti-α-actinin. Anti-zipper antibody was used to assess the level of *zipper* knock-down. (B) Primary muscle cells were isolated from *zip^p1215^/Cyo-GFP* and *zipper* mutant embryos identified by the lack of GFP expression. Cultures were stained with anti-MHC, anti-actin and anti-α-actinin. Scale bars: 10 µm.
(2.87 MB TIF)

Figure S10 Zipper/Zasp/α-actinin senses Ca^2+^ stress in vitro. (A) Various amounts of dsRNAs against *PMCA* were added to primary muscle cells. Muscle striation was monitored by anti-MHC and anti-actin antibodies. 50 ng of *PMCA* is sufficient to induce muscle phenotypes characteristic of sarcomere disruption. (B) Different combinations of 40 ng dsRNA against *PMCA* with 250 ng dsRNA against *Zasp* or *zipper* or *α-actinin* were applied to primary muscles followed by anti-MHC and anti-actin staining. Scale bars: 10 µm.
(7.09 MB TIF)

Figure S11 Tension sensor components are still localized at muscle ends in the paralyzed animals. Confocal micrographs of late embryonic body wall muscles from a control animal (top panels), and age comparable muscles from *imac* null mutant (bottom panels) stained for MHC (green in merge), α-actinin (blue in merge) and Zasp (red in merge). Scale bar: 10 µm. Note that the tension sensor components Zasp and α-actinin were still localized at muscle attachment sites (arrows in the bottom panels) even though the sarcomere structures were disrupted in *imac* null mutant muscles.
(3.61 MB TIF)

Figure S12 Titin is crucial for sarcomere assembly. A series of antibodies against MHC, actin, TnT, TnI, TnC, Tm, α-actinin, Zasp, zipper, Mlp84B, spectrin were used to detect muscle striation. Anti-kettin/titin was applied to assess the knock-down efficacy. Scale bars: 10 µm.
(4.24 MB TIF)
