# Supplementary figures and images for: Sarcomere Formation Occurs by the Assembly of Multiple Latent Protein Complexes
Source: PLoS Genet. 2010 Nov 18;6(11):e1001208. doi: 10.1371/journal.pgen.1001208 (PMC2987826; doi:10.1371/journal.pgen.1001208)

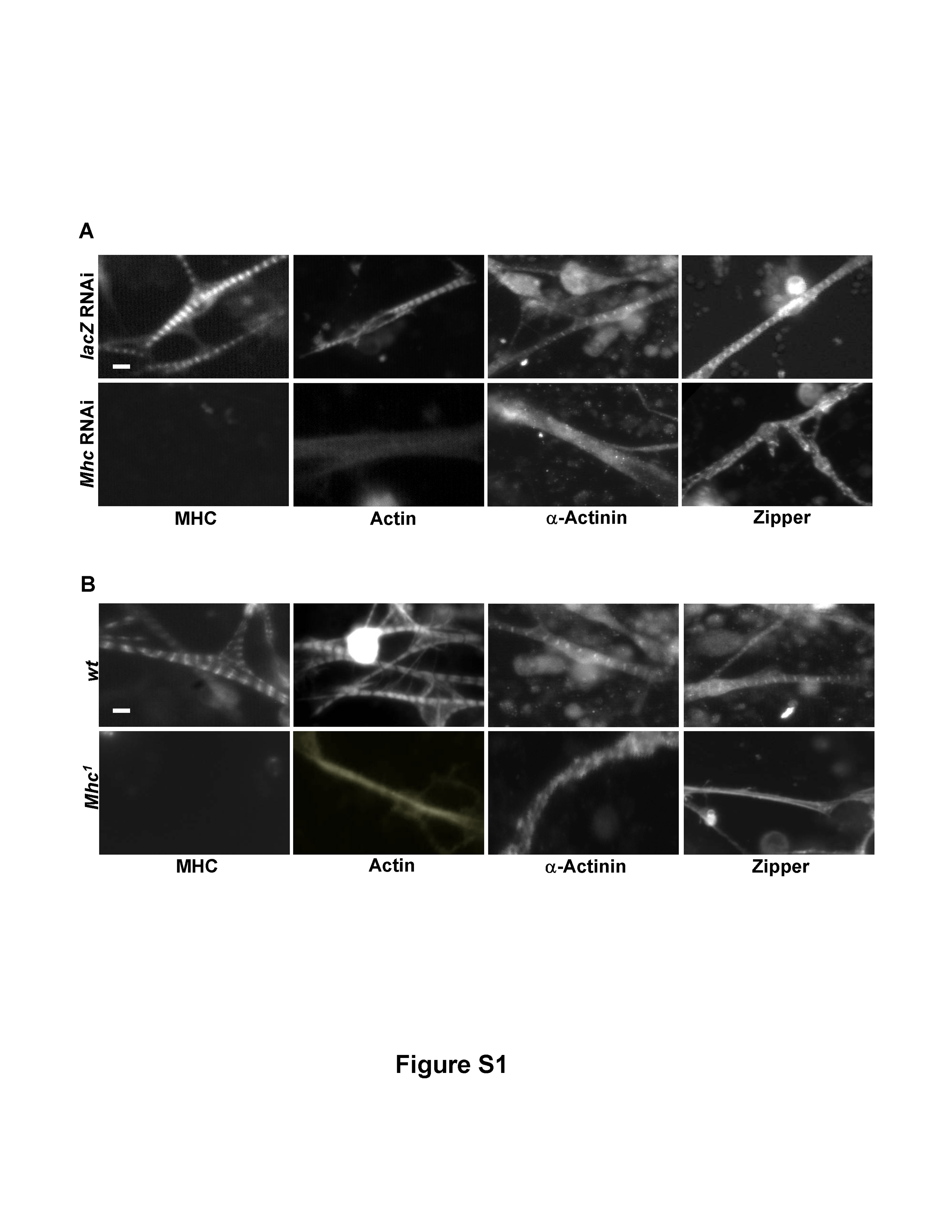

Supplement: Figure S1 — MHC is required for sarcomere formation. (A,B) Primary muscle culture cells were isolated from Mhc-GFP and treated with Mhc dsRNA. (A) Mhc1 mutant embryos. (B) Anti-MHC staining was used to assess the knock-down efficiency or to identify Mhc mutant primary muscle cells. Cultures were immunostained using anti-actin or anti-α-actinin to analyze sarcomeric structures. Anti-zipper antibody was used to analyze the localization of zipper in the absence of MHC. Scale bars: 10 µm. (2.20 MB TIF) [file pgen.1001208.s001.tif]

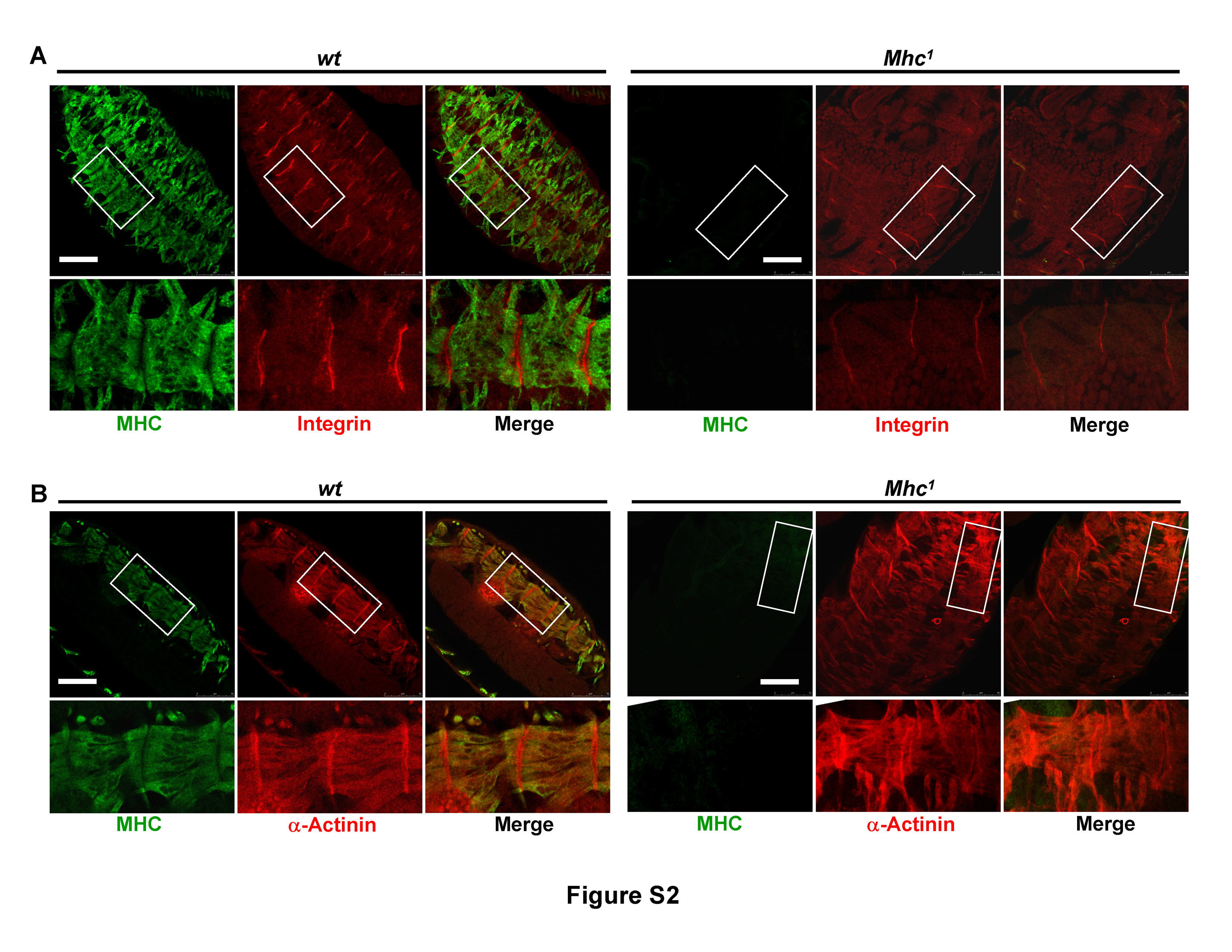

Supplement: Figure S2 — Removal of MHC has no effect on the localization of integrin and α-actinin. (A,B) Staining of integrin and α-actinin was performed in both wild-type and Mhc1 mutant stage 15–16 embryos. MHC antibody was used to analyze Mhc1 null allele. Both MHC and other sarcomeric protein stainings were merged to check their localization relationships. Scale bar: 20 µm. (7.76 MB TIF) [file pgen.1001208.s002.tif]

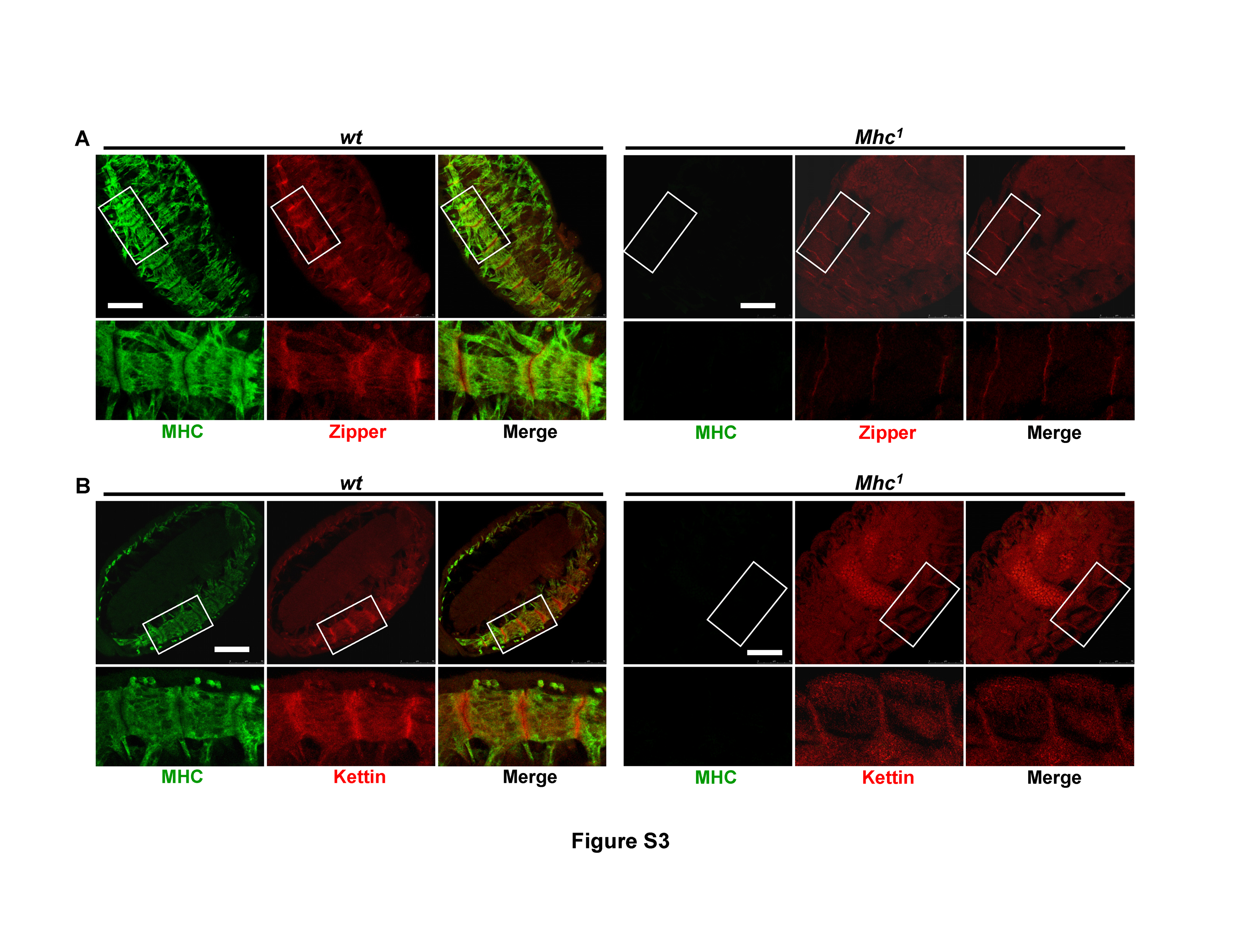

Supplement: Figure S3 — Localization of zipper and kettin at muscle attachment sites in the absence of MHC. (A,B) Zipper and kettin were stained with antibodies to show their muscle attachment site localization in the presence and absence of MHC in stage 15–16 embryos. Scale bar: 20 µm. (6.09 MB TIF) [file pgen.1001208.s003.tif]

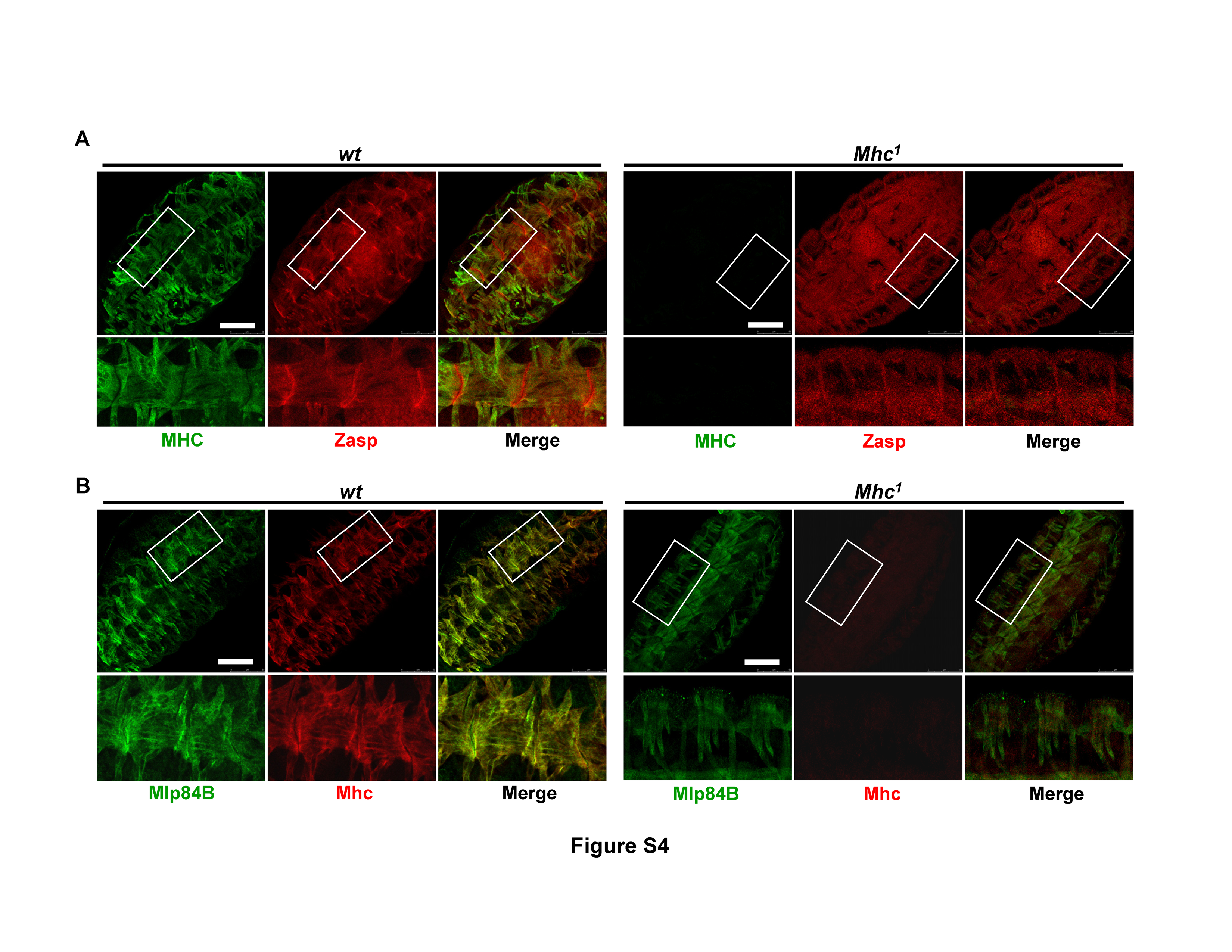

Supplement: Figure S4 — Zasp and Mlp84B localize at muscle attachment site in a MHC-independent manner. (A,B) Zasp and Mlp84B distribution were assessed by their antibody stainings and merged with MHC staining to check their localization relationships in 15–16 stage embryos. Scale bars: 20 µm. (6.41 MB TIF) [file pgen.1001208.s004.tif]

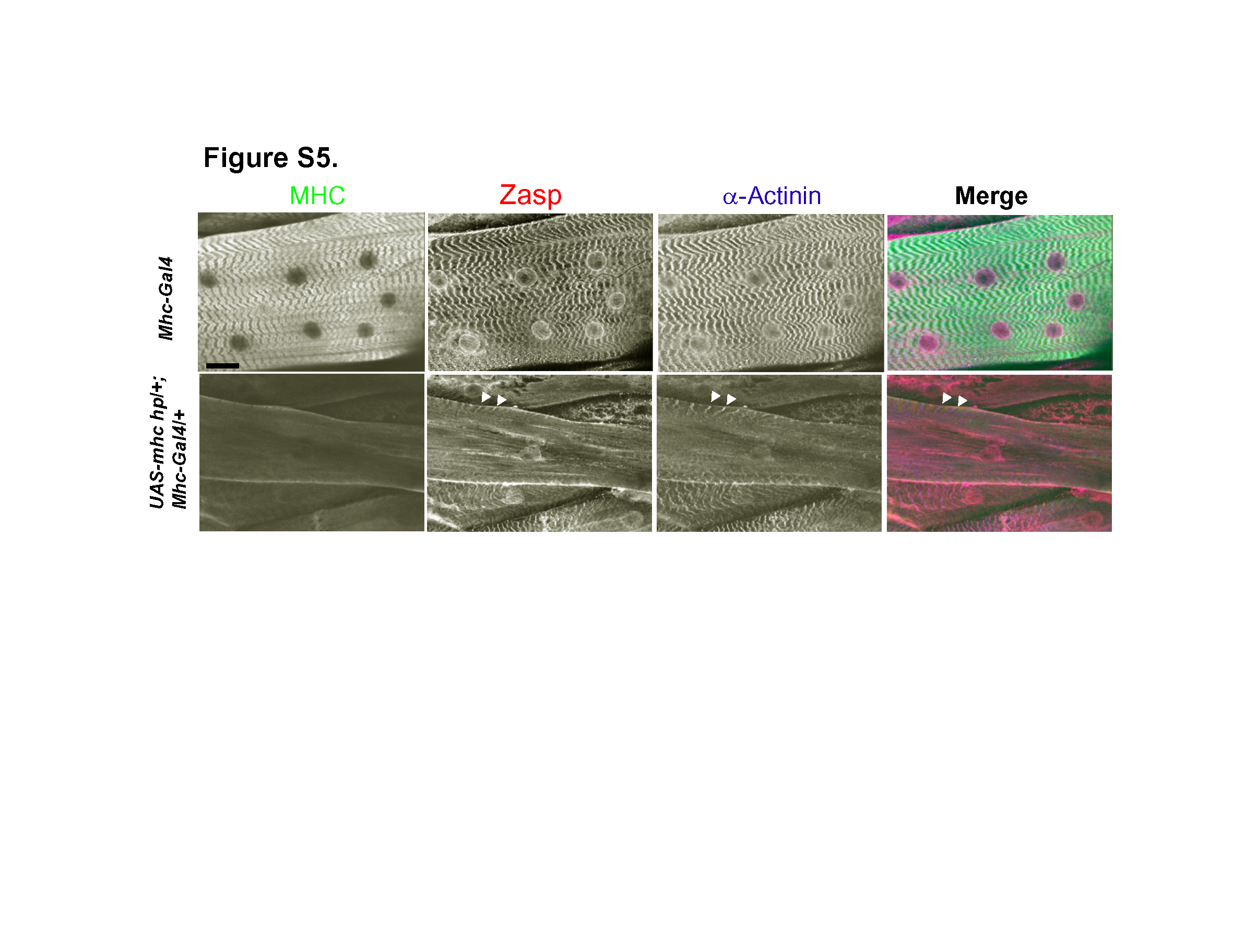

Supplement: Figure S5 — Localization of sarcomeric components in larval muscles is disrupted upon MHC reduction. Confocal micrographs of second instar larval body wall muscles from control animal (top panels) and age comparable muscles from a larva carrying transgenes of Mhc-Gal4;UAS-Mhc hp (bottom panels) stained for MHC (green in merge), α-actinin (blue in merge) and Zasp (red in merge). Scale bar: 50 µm. Note that the presence of striated organization of these sarcomeric components correlated well with the presence of MHC expression (arrowheads at bottom panels), while loss of MHC expression led to disruption of sarcomere striation and distribution of these sarcomeric proteins. (2.73 MB TIF) [file pgen.1001208.s005.tif]

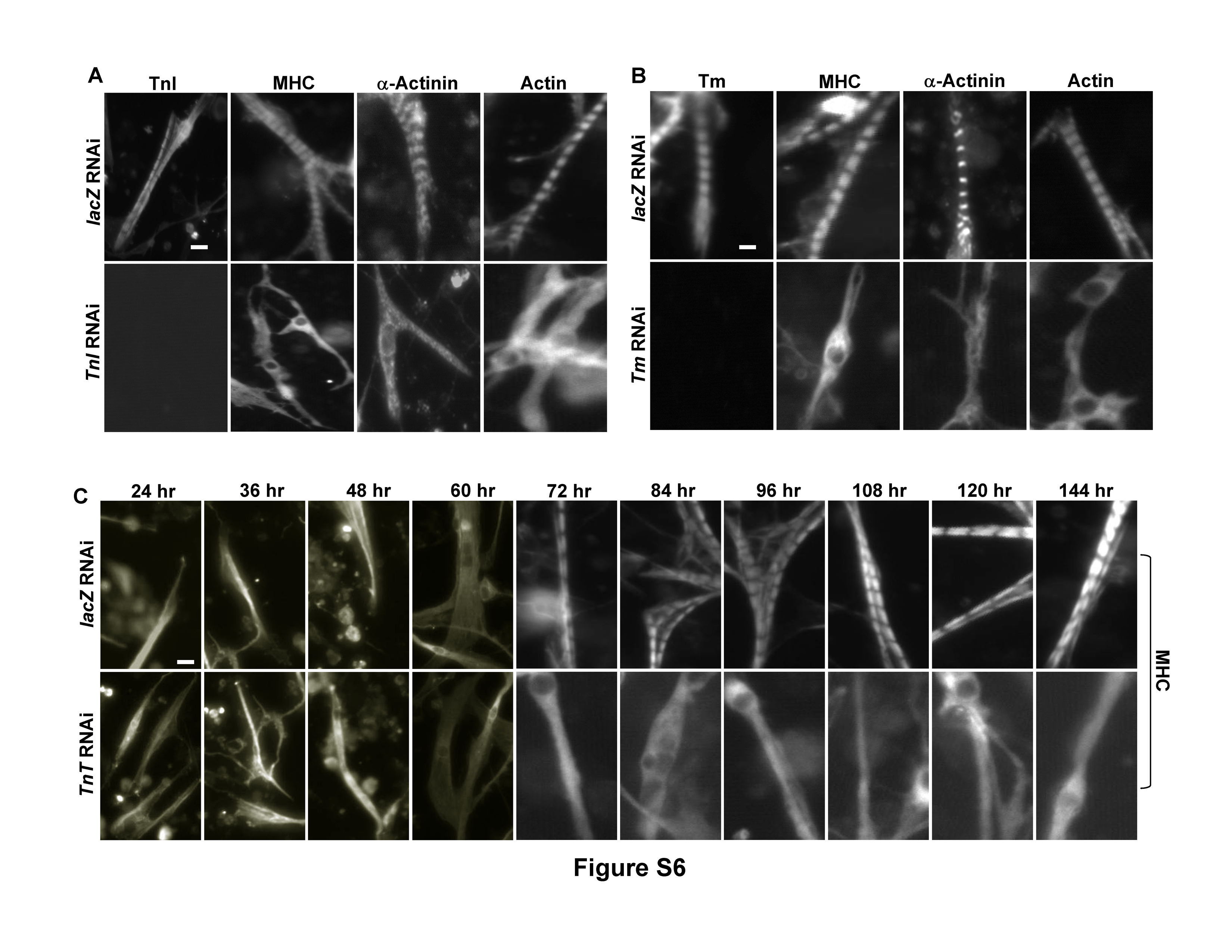

Supplement: Figure S6 — The Tn-Tm complex is essential for sarcomere assembly. (A,B) DsRNAs against TnI or Tm were applied to primary muscle cells, and anti-TnI and anti-Tm antibodies were used to document the knock-down effectiveness. The sarcomeric organization of treated muscles was analyzed using anti-actin and anti-α-actinin antibodies. (C) TnT knock-down time course experiment. No striation was observed in TnT RNAi-treated primary muscle cells, even at 3 days after plating when the sarcomeres begin to form in the lacZ RNAi control. Muscle cultures were stained with anti-MHC antibody. Scale bars: 10 µm. (3.84 MB TIF) [file pgen.1001208.s006.tif]

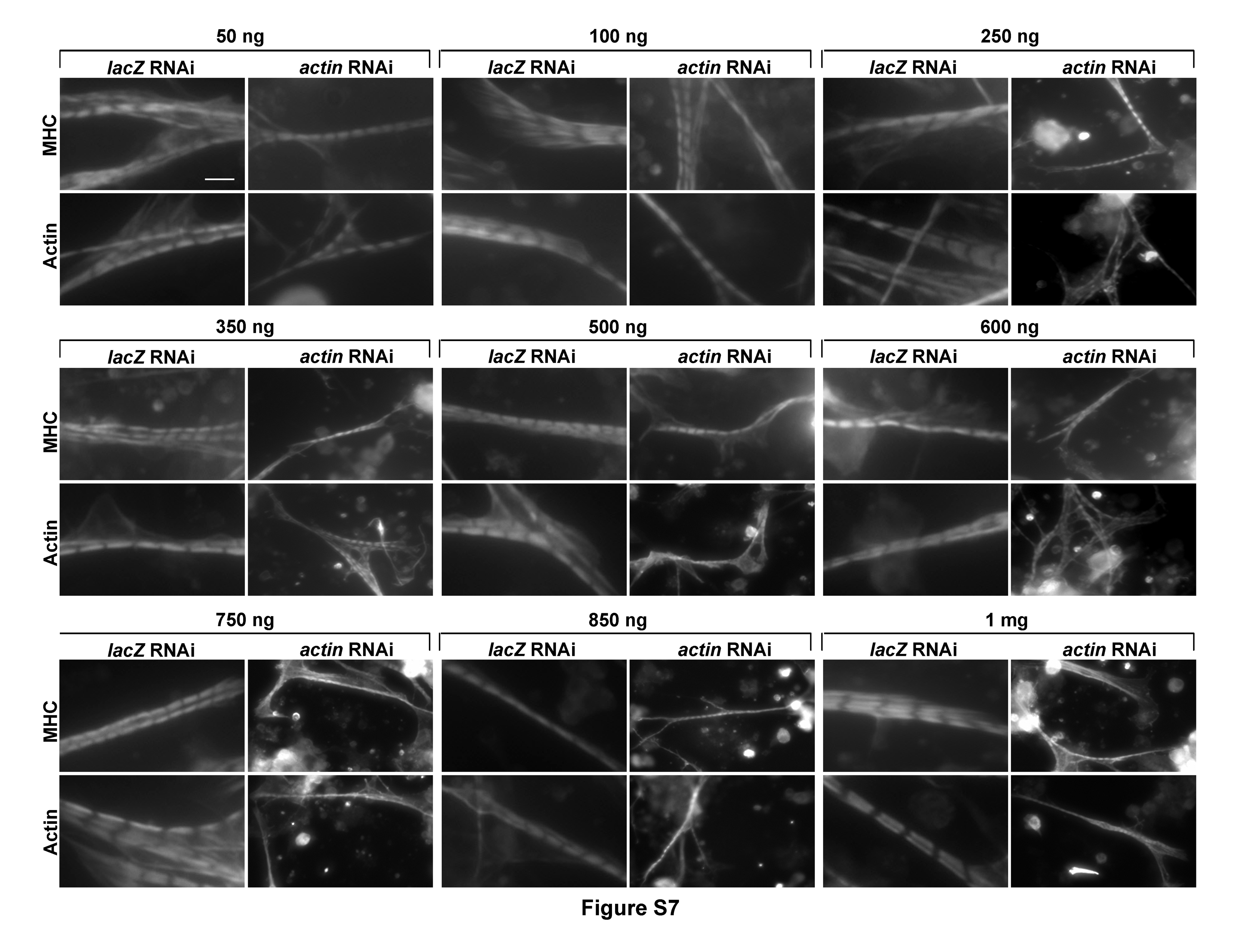

Supplement: Figure S7 — Persistent arrest of residual actin protein in myofibril after dsRNA treatment. Different amounts of actin dsRNA were added to primary muscle cell culture from 50 ng to 1 mg. lacZ dsRNA was used as a control. Anti-actin antibody was applied for analysis of residual actin signal and anti-MHC for muscle structure. Scale bars: 10 µm. (3.38 MB TIF) [file pgen.1001208.s007.tif]

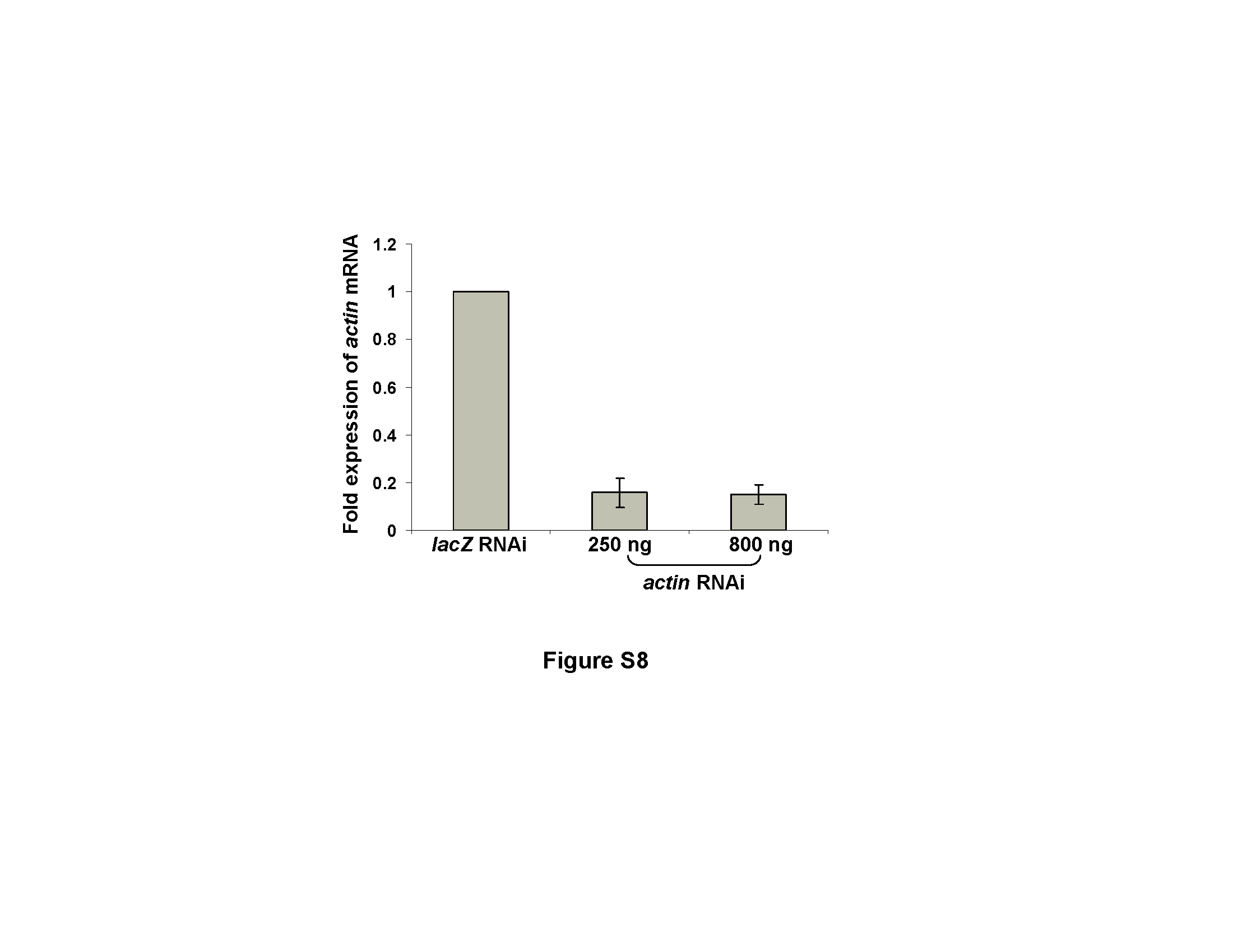

Supplement: Figure S8 — Quantitative RT-PCR analysis of actin RNAi efficiency. 250 ng or 800 ng of dsRNA against actin were applied to Drosophila S2 cells in comparison of treatment with 250 ng of lacZ dsRNA. Quantitative RT-PCR analysis was performed to assess the actin knock-down effectiveness. The amount of actin mRNA from lacZ dsRNA treatment was used as a normalization control. (0.27 MB TIF) [file pgen.1001208.s008.tif]

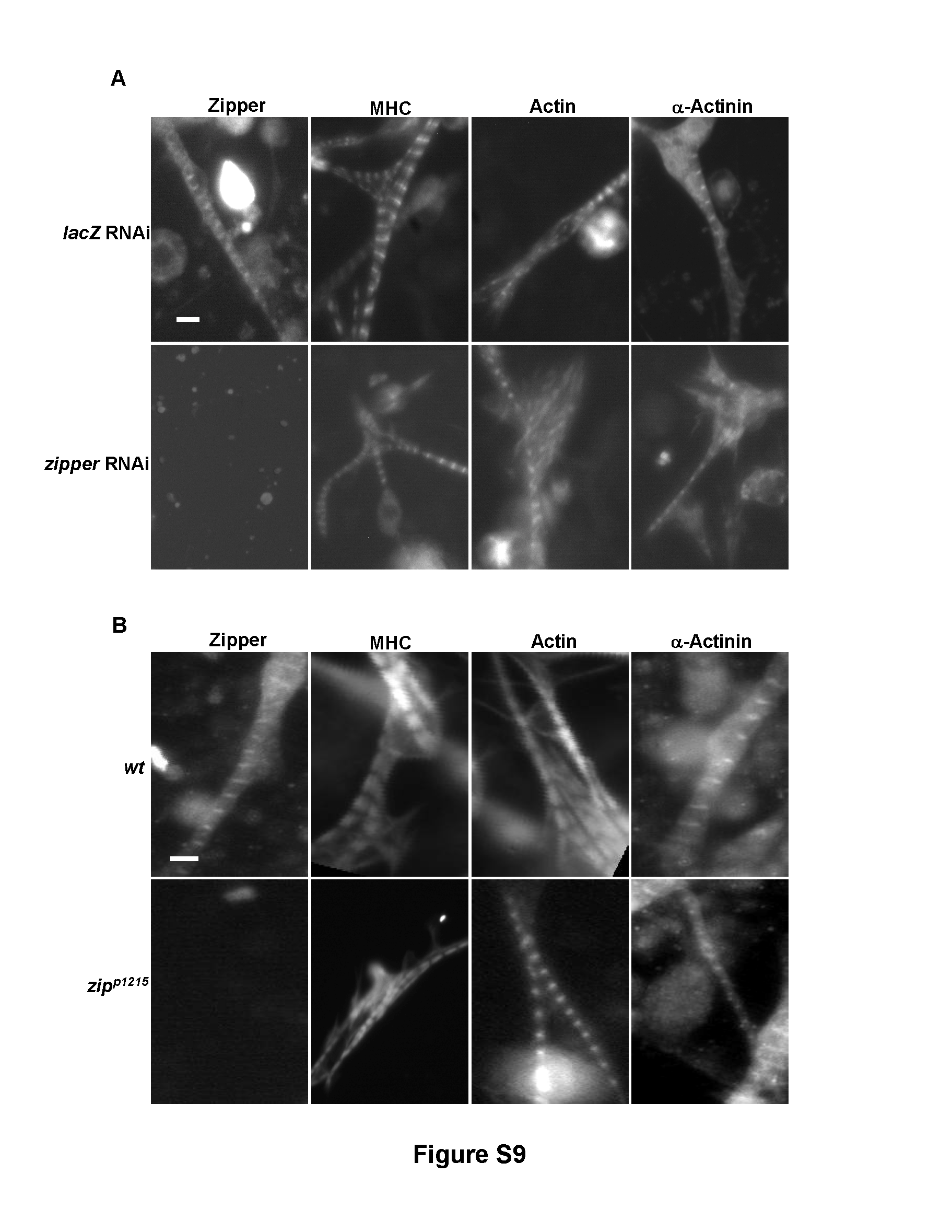

Supplement: Figure S9 — Zipper is not exclusively required for sarcomere striation. (A) Primary muscle cells treated with zipper dsRNA and stained using anti-MHC, anti-α-actinin. Anti-zipper antibody was used to assess the level of zipper knock-down. (B) Primary muscle cells were isolated from zipp1215/Cyo-GFP and zipper mutant embryos identified by the lack of GFP expression. Cultures were stained with anti-MHC, anti-actin and anti-α-actinin. Scale bars: 10 µm. (2.87 MB TIF) [file pgen.1001208.s009.tif]

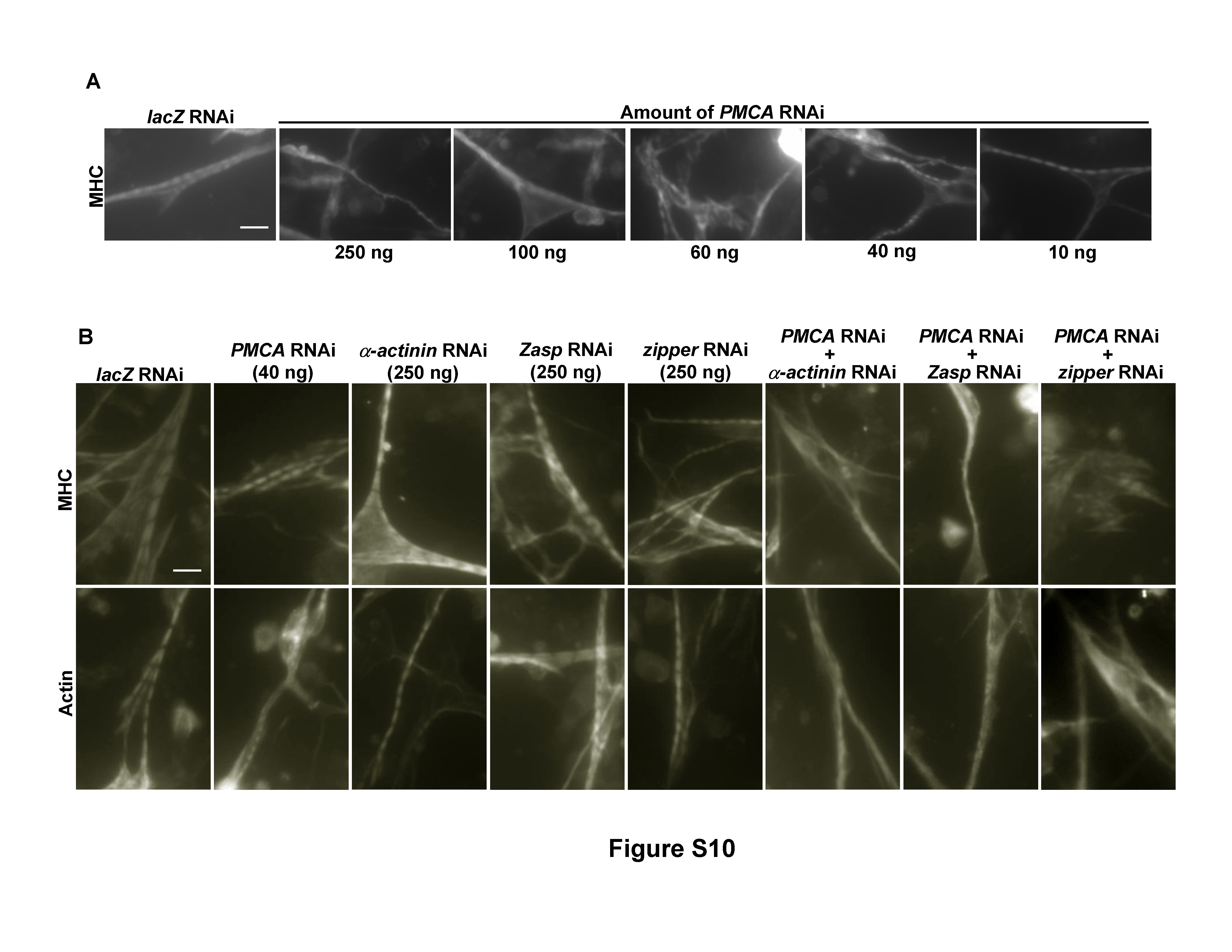

Supplement: Figure S10 — Zipper/Zasp/α-actinin senses Ca2+ stress in vitro. (A) Various amounts of dsRNAs against PMCA were added to primary muscle cells. Muscle striation was monitored by anti-MHC and anti-actin antibodies. 50 ng of PMCA is sufficient to induce muscle phenotypes characteristic of sarcomere disruption. (B) Different combinations of 40 ng dsRNA against PMCA with 250 ng dsRNA against Zasp or zipper or α-actinin were applied to primary muscles followed by anti-MHC and anti-actin staining. Scale bars: 10 µm. (7.09 MB TIF) [file pgen.1001208.s010.tif]

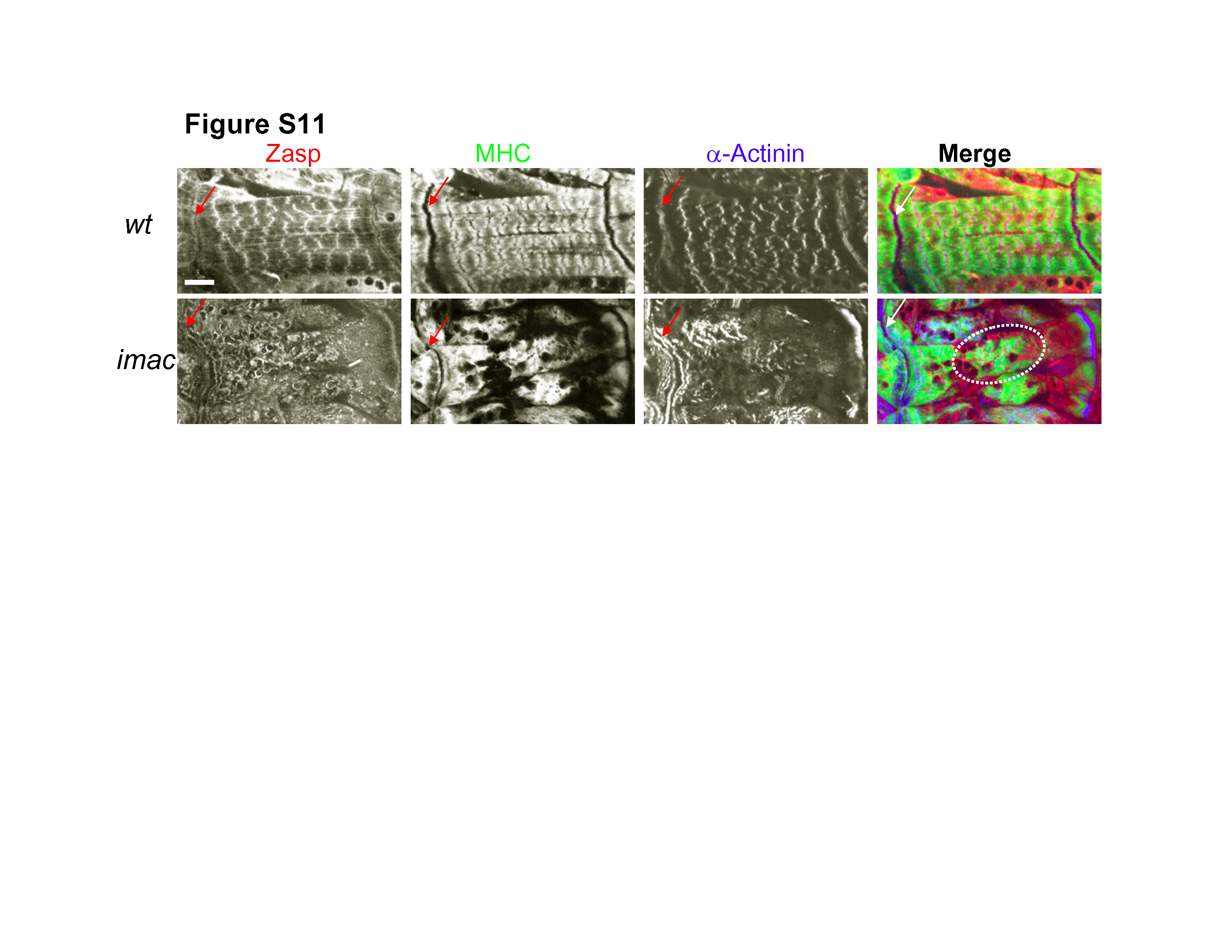

Supplement: Figure S11 — Tension sensor components are still localized at muscle ends in the paralyzed animals. Confocal micrographs of late embryonic body wall muscles from a control animal (top panels), and age comparable muscles from imac null mutant (bottom panels) stained for MHC (green in merge), α-actinin (blue in merge) and Zasp (red in merge). Scale bar: 10 µm. Note that the tension sensor components Zasp and α-actinin were still localized at muscle attachment sites (arrows in the bottom panels) even though the sarcomere structures were disrupted in imac null mutant muscles. (3.61 MB TIF) [file pgen.1001208.s011.tif]

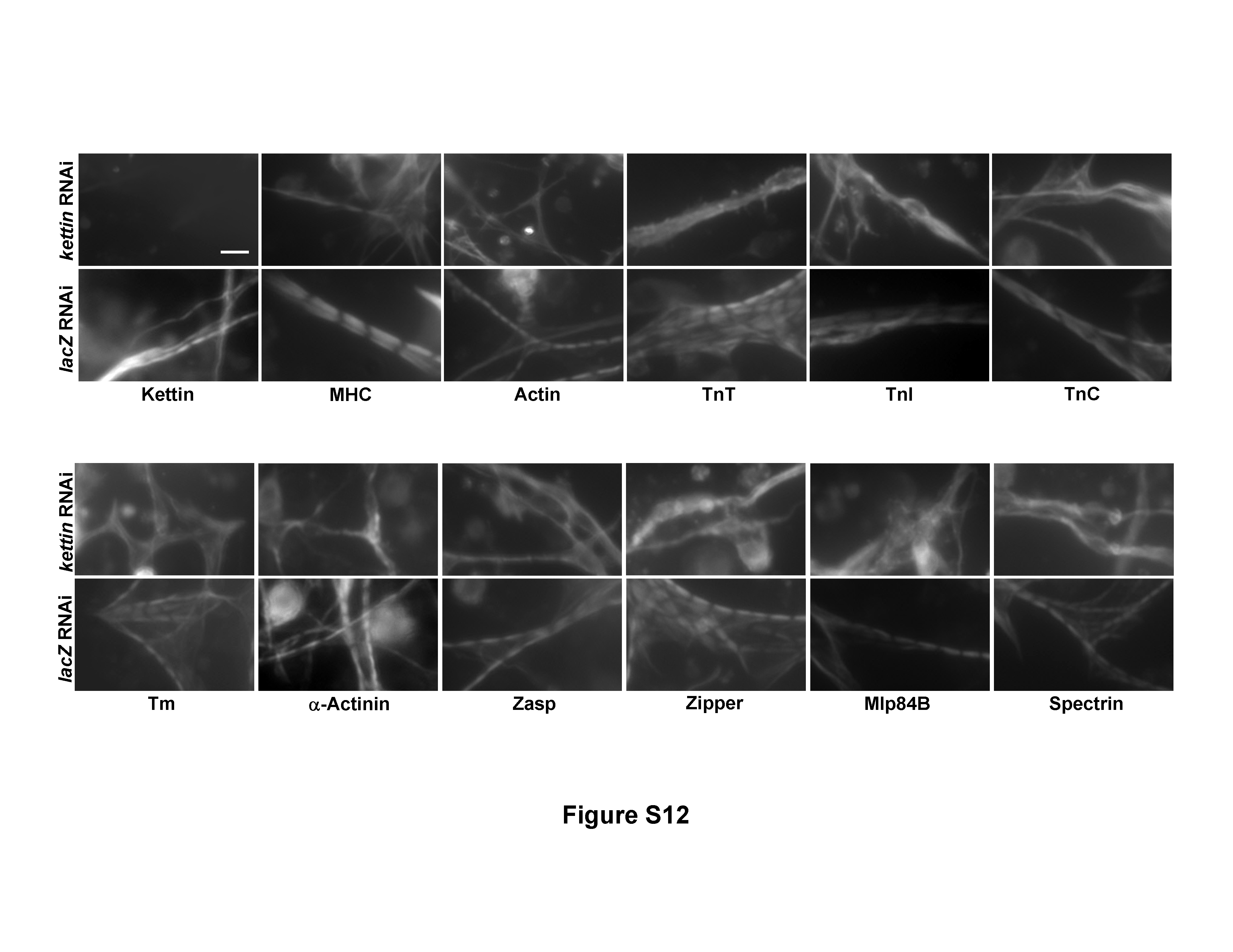

Supplement: Figure S12 — Titin is crucial for sarcomere assembly. A series of antibodies against MHC, actin, TnT, TnI, TnC, Tm, α-actinin, Zasp, zipper, Mlp84B, spectrin were used to detect muscle striation. Anti-kettin/titin was applied to assess the knock-down efficacy. Scale bars: 10 µm. (4.24 MB TIF) [file pgen.1001208.s012.tif]
